# Basic clinical features do not predict dopamine transporter binding in idiopathic REM behavior disorder

**DOI:** 10.1038/s41531-018-0073-1

**Published:** 2019-01-29

**Authors:** L. M. Chahine, A. Iranzo, A. Fernández-Arcos, T. Simuni, N. Seedorff, C. Caspell-Garcia, A. W. Amara, C. Comella, B. Högl, J. Hamilton, K. Marek, G. Mayer, B. Mollenhauer, R. Postuma, E. Tolosa, C. Trenkwalder, A. Videnovic, W. Oertel, the PPMI Sleep Working Group, B. Kumar, L. James, G. Nomikos, J. Cedarbaum, M. Yang, M. Brys, V. Irzhevesky, K. Schmidt, N. Jennings, A. Reith, D. Tattersall, M. Sanchez, N. Daegele, C. Min, R. Malkani, J. Peterschmitt, P. Sardi, S. Bozzi, T. Fischer, R. Evans, V. Kiyasova, A. Simen, A. Siderowf

**Affiliations:** 1 Department of Neurology, The University of Pittsburgh, Pittsburgh, PA USA; 2 Neurology Service, Hospital Clinic de Barcelona, IDIBAPS, CIBERNED, Barcelona, Spain; 3 Department of Neurology, Feinberg School of Medicine, Northwestern University, Chicago, IL USA; 4 Department of Biostatistics, The University of Iowa, Iowa City, IA USA; 5 Department of Neurology, The University of Alabama at Birmingham, Birmingham, AL USA; 6 Department of Neurology, Rush University, Chicago, IL USA; 7 Department of Neurology, Innsbruck Medical University, Innsbruck, Austria; 8 The Michael J. Fox Foundation for Parkinson’s Research, New York, NY USA; 9 Institute for Neurodegenerative Disorders, New Haven, CT USA; 10 Department of Neurology, Hephata-Klinik, Hephata Hessisches Diakoniezentrum, e.V, Weibersbrunn, Germany; 11 Department of Neurology, University Medical Center, Göttingen, Germany; 12 Paracelsus-Elena-Klinik, Kassel, Germany; 13 Division of Neurology, McGill University, Montreal, QC Canada; 14 Department of Neurology, Massachusetts General Hospital, Boston, MA USA; 15 Department of Neurology, Philipps University, Marburg, Germany; 16 Charitable Hertie Foundation, Frankfurt/Main, Germany; 17 AbbVie Inc., Chicago, IL USA; 18 Allergan, Irvine, CA USA; 19 Biogen Idec, Cambridge, MA USA; 20 Celgene, Summit, NJ USA; 21 GlaxoSmithKline, Brentford, UK; 22 Merck & Co, Kenilworth, NJ USA; 23 Sanofi, Paris, France; 24 Servier, Neuilly-sur-Seine, France; 25 Takeda, Tokyo, Japan; 26 University of Pennsylvania, Philadelphia, PA USA

## Abstract

REM sleep behavior disorder (RBD) is strongly associated with development of Parkinson’s Disease and other α-synuclein-related disorders. Dopamine transporter (DAT) binding deficit predicts conversion to α-synuclein-related disorders in individuals with RBD. In turn, identifying which individuals with RBD have the highest likelihood of having abnormal DAT binding would be useful. The objective of this analysis was to examine if there are basic clinical predictors of DAT deficit in RBD. Participants referred for inclusion in the RBD cohort of the Parkinson Progression Markers Initiative were included. Assessments at the screening visit including DAT SPECT imaging, physical examination, cognitive function screen, and questionnaire-based non-motor assessment. The group with DAT binding deficit (*n* = 49) was compared to those without (*n* = 26). There were no significant differences in demographic or clinical features between the two groups. When recruiting RBD cohorts enriched for high risk of neurodegenerative disorders, our data support the need for objective biomarker assessments.

## Sleep disorders: Looking for predictive signs of neurodegenerative disease

The clinical characteristics of patients with rapid eye movement sleep behavior disorder (RBD) are not associated with reduced dopamine transporter binding, an established imaging biomarker of Parkinson’s Disease (PD). Because around 80 percent of patients with RBD develop PD, there is great hope that research on these patients will help uncover early signs of the disease and guide the development of neuroprotective therapies. Lana Chahine at The University of Pittsburgh, USA, and colleagues in the Parkinson Progression Markers Initiative (PPMI) Sleep Working Group analyzed the clinical features of 75 individuals with RBD. They found no significant differences in demographic features or in motor and non-motor symptoms between RBD patients with dopamine transporter binding deficit and those without. These findings highlight the need to assess dopamine transporter binding to determine the future risk of PD.

## Introduction

Rapid eye movement (REM) sleep behavior disorder (RBD) is one of the strongest pre-motor clinical markers of future development of Parkinson’s disease (PD) and other α-synuclein-related disorders.^1^ Indeed, a neurodegenerative α-synuclein-related disorder such as PD, dementia with Lewy bodies (DLB), or multiple system atrophy (MSA) develops in the majority of individuals with idiopathic RBD (iRBD).^2^ As such, individuals with iRBD can be targets for future disease-modification and neuroprotective therapies to prevent synucleinopathies. However, there are a subset of RBD patients who remain free of a neurodegenerative disorder for prolonged periods of time (years to even decades). In addition, there are several clinical mimics of RBD (including, but not limited to, obstructive sleep apnea (OSA), post-traumatic stress disorder (PTSD), and non-REM parasomnias). Including individuals from the latter two groups in clinical trials aimed at preventing or delaying progression to a diagnosable synucleinopathy could be detrimental. Thus, biomarkers to predict which patients have higher risk of developing a neurodegenerative synucleinopathy, and when this will occur, are needed. Several clinical features, including various motor and non-motor signs and symptoms,^3,4^ as well as imaging findings such as reduced dopamine transporter (DAT) binding on single-photon emission computed tomography (SPECT) scan^5^ have been reported to predict emergence of a neurodegenerative syndrome. Since DAT SPECT is costly and associated with radiation exposure (albeit small), a two-tiered approach has been advocated, whereby low cost assessments, alone or in combination, are used to identify individuals who have the highest likelihood of having an abnormal DAT SPECT.^6^ Ultimately, this information would inform resource utilization and enrollment criteria for future observational studies and clinical trials for RBD and other prodromal PD states. Clinical predictors of DAT binding deficit that could be easily and routinely ascertained by clinicians would be particularly valuable.

The Parkinsons Progression Markers Initiative (PPMI) RBD cohort was assembled with the goal of identifying biomarkers to inform future risk of clinically and pathologically defined neurodegenerative syndromes. As part of the screening process for this study, individuals with a clinical diagnosis of iRBD were referred for DAT SPECT. The objective of this analysis was to examine whether there are basic clinical predictors of DAT binding deficit in this patient population i.e., low-cost, routinely available and/or easily ascertainable predictors.

## Results

### Comparison of demographic, motor, and non-motor features

Table 1 shows demographic features of the RBD cohort screened for participation in PPMI. There were no significant differences in the iRBD group with DAT binding deficit vs those without DAT binding deficit on any of the examined demographic features (Table 1). Table 2 shows motor and non-motor signs and symptoms in the iRBD group with DAT binding deficit compared to the iRBD group without DAT binding deficit. In univariate analyses, there were no significant differences in motor and non-motor symptoms or cognitive function between the groups. Using a cutoff on the MDS-UPDRS items of >1 vs. >0 did not change these results (supplementary table 1).

**Table 1:** Demographic characteristics of individuals referred with a clinical diagnosis of iRBD with vs. without DAT binding deficit

	DAT binding deficit present (*n* = 49)	No DAT binding deficit (*n* = 26)	*p*-value for differences between groups
Mean age in years (SD; Min; Max)	69.84 (6.2; 51.0, 84.7)	69.38 (7.7; 60.0, 86.3)	0.7774
Male: Female *n* (%)	40 (81.63):9 (18.37)	19 (73.08):7 (26.92)	0.3894
Education			
<13 Years *n* (%)	20 (40.82)	15 (57.69)	0.1633
≥ 13 Years *n* (%)	29 (59.18)	11 (42.31)	
Family history of PD			0.8981
Yes *n* (%): No *n* (%)	7 (14.29):42 (85.71)	4 (15.38):22 (84.62)	
Handedness right *n* (%): Left or mixed *n* (%)	44 (89.80):5 (10.20)	24(92.31):2 (7.69)	0.7219
Mean RBD disease duration from diagnosis in years (SD; Min; Max)^b^	2.85 (3.1; 0.1–11.8)	3.58 (3.4; 0–9.9)	0.3615
Mean RBD disease duration from symptom onset in years (SD; Min; Max)^b^	9.61 (6.8; 0.38–30.26)	3.58 (3.4; 0.03–9.90)	0.6484
REM sleep without atonia on polysomnogram confirmed by the PPMI sleep core^a^, *n* (%)	41 (97.62)	20 (95.24)	0.6114

^a^Twelveparticipants did not have polysomnographic data available for review by the sleep core (in these cases, polysomnogram had been performed and showed REM sleep without atonia, but the raw polysomnographic data were not available for tranfer to the central sleep core for confirmation). Seven of these cases were in the DAT Deficit+ group and 5 in the DAT Deficit− group

^b^RBD disease and symptom duration was missing on two subjects

**Table 2:** Motor and non-motor signs and symptoms in the iRBD group with DAT binding deficit compared to the iRBD without DAT binding deficit

	DAT binding deficit present (*n* = 49)	No DAT binding deficit (*n* = 26)	*p*-value for differences between groups
MDS-UPDRS III total score mean (SD; Min, Max)	4.51 (3.8; 0–15)	4.15 (3.9; 0–13)	0.7023
MDS-UPDRS III bradykinesia subscore > 0, *n* (%)	17 (34.69%)	10 (38.46%)	0.8033
MDS-UPDRS III tremor subscore > 0, *n* (%)	18 (36.73%)	10 (38.46%)	1.0000
MDS-UPDRS III rigidity subscore > 0, *n* (%)	10 (20.41%)	6 (23.08%)	0.7758
MDS-UPDRS item 1.1: cognitive Impairment score > 0, *n* (%)	20 (40.82)	12 (46.15)	0.8067
MDS-UPDRS item 1.2: hallucinations score > 0, *n* (%)	1 (2.04)	2 (7.69)	0.2743
MDS-UPDRS item 1.3: depression score > 0, *n* (%)	11 (22.45)	9 (34.62)	0.2829
MDS-UPDRS item 1.4; anxiety score > 0, *n* (%)	16 (32.65)	9 (34.62)	1.0000
MDS-UPDRS item 1.5: apathy score > 0, *n* (%)	7 (14.29)	1 (3.85)	0.2490
MDS-UPDRS item 1.7: Nocturnal sleep abnormalities score > 0, *n* (%)	41 (83.67)	24 (92.31)	0.4784
MDS-UPDRS item 1.8 daytime sleepiness score > 0, *n* (%)	36 (73.47)	19 (73.08)	1.0000
MDS-UPDRS item 1.13: fatigue score > 0, *n* (%)	28 (57.14)	11 (42.31)	0.2368
MDS-UPDRS item 1.11: constipation score > 0, *n* (%)	23 (46.94)	15 (57.69)	0.4686
Orthostatic hypotension present, *n* (%)	25 (51.02)	8 (30.77)	0.0927
MOCA mean (SD; Min, Max)	25.27 (4.2; 11.0, 30.0)	24.88 (4.0; 16.0, 30.0)	0.7039
MOCA word generation mean (SD; Min, Max)	13.18 (5.2; 1.0, 26.0)	13.23 (6.0; 2.0, 32.0)	0.9717
MOCA delayed recall mean (SD; Min, Max)	3.06 (1.4; 0.0, 5.0)	2.85 (1.6; 0.0, 5.0)	0.5563
Visuospatial total score mean (SD; Min, Max)	3.88 (1.2; 0.0, 5.0)	3.88 (1.1; (1.0, 5.0)	0.9802

In a logistic regression model examining predictors of DAT binding deficit, none were identified. In linear regression models examining DAT binding as a continuous variable (mean striatum specific binding ratio (SBR)) in the entire sample, there were no significant associations with any of the measures of motor or non-motor function (supplementary table 3; similarly there were no significant associations when the outcome variable was DAT binding as a continuous variable in the right, left, or mean caudate or putamen).

## Discussion

In this study, we examined predictors of DAT binding in individuals with a clinical diagnosis of iRBD, recruited from clinics at tertiary care centers in the USA and Europe. We did not find that any of the examined demographic, motor, or non-motor features were predictive of DAT binding deficit in individuals with a clinical diagnosis of iRBD.

In longitudinal studies of individuals with iRBD at baseline who are otherwise free of a neurologic diagnosis, several clinical and biomarker features predictive of eventual diagnosis of a neurodegenerative parkinsonian syndrome have been identified. Clinical signs and symptoms include olfactory dysfunction,^4^ impaired color vision,^4^ abnormalities on objective motor testing,^7^ orthostatic hypotension,^8^ urinary dysfunction,^8^ and constipation,^8^ and these have been incorporated into research criteria for prodromal PD.^1^ However, these clinical signs/symptoms have low specificity in isolation, and in identifying individuals at future risk of PD, a two-tiered approach has been advocated for, whereby low cost, minimal risk assessments are used to screen large numbers for possible prodromal risk, and more costly studies with potentially higher risk are used as a 2nd test with higher specificity.^6^ Our analysis extends this concept to examine predictors not of future risk of PD, but cross-sectional DAT binding deficit on SPECT.

There is accumulating evidence that reduced DAT binding, a reflection of striatal dopaminergic denervation, predicts a high future risk of PD in individuals with prodromal clinical features such as iRBD, hyposmia, or symptoms of autonomic dysfunction.^5,9^ While a normal DAT SPECT scan does not eliminate risk of future PD, it is likely that individuals with a normal DAT SPECT are at a lower risk over the next few years of developing PD compared to those with an abnormal scan. In that context, the main implications for our findings pertain to observational studies and clinical trials aiming to identify individuals who are considered prodromal or at-risk for PD. In studies attempting to recruit individuals with incipient neurodegeneration (e.g., a cohort enriched with individuals at greatest risk of PD or another neurodegenerative synucleinopathy), a clinical diagnosis of iRBD in isolation or in combination with easily-ascertainable, routinely collected motor and non-motor features may be insufficient. Rather, additional objective biomarker evidence of neurodegeneration would be required. While the current analysis does not aim to test the predictive utility of abnormal DAT binding or other biomarkers for future risk of phenoconversion to a neurodegenerative disorder, longitudinal follow-up of the prodromal PPMI cohort will further inform which clinical, imaging, and biofluid biomarkers, alone or in combination, are most predictive of that future risk.

Several limitations of this study warrant mention. There are limited assessments administered at the screening visit of the PPMI study, and thus several prodromal markers were not assessed including olfaction and autonomic function. In addition, at the screening visit, only a global measure of cognition, namely the MoCA, was obtained, and our analysis of specific cognitive domains was thus limited to MoCA subscores. This, along with our relatively small sample size, may have limited our ability to detect significant differences between the groups, and increases the chances of a type 2 error. Furthermore, what assessments were administered were developed and validated in manifest PD, and their utility in detecting signs and symptoms in iRBD is not known. Thus, caution is warranted in interpretation of our results. Despite these limitations, we believe that our results are still of relevance to recruiting iRBD research participants from the clinic, where assessments during routine clinical care are often limited to the types of variables examined in this analysis.

## Methods

### Study participants

PPMI is a multicenter international prospective cohort study. Study aims, methodology, and details of study assessments are available on the PPMI website (http://www.ppmi-info.org/study-design). Subjects considered in this analysis were individuals referred to undergo a screening visit for possible participation in the PPMI iRBD cohort (*n* = 75). Inclusion criteria for screening included a clinical diagnosis of polysomnographically-confirmed iRBD made by a physician (most often a Movement Disorders or Sleep Specialist), with confirmation by the site investigator of the diagnosis based on clinical grounds (obtaining a comprehensive clinical history and physical examination). Exclusion criteria were a diagnosis of Parkinson’s disease, dementia, or any other neurological disorder as determined by the investigator.

In order for subjects to be considered for the study, they had to have a history of REM without atonia (RWA) on polysomnogram (PSG). These PSGs were performed as part of the patient’s clinical evaluation by their treating provider, and the protocol for their acquisition varied based on the practices of the sleep lab at which they were acquired. Therefore, criteria for determination of RWA for clinical diagnosis were not standardized but rather based on the individual clinical sleep lab’s criteria for determination of RWA. In addition, in cases where the clinical sleep lab PSG was still archived and could be obtained (*n* = 63), the PSGs were transferred to the PPMI sleep core for further interpretation and to confirm the presence of RWA based on pre-defined criteria, as follows. The sleep core made a determination of RWA based on evaluation of surface EMG recordings from the mentalis muscle on PSG. REM sleep without atonia was defined based on the following criteria^10^ (i) In 3 s bins: 18% increase in tonic and/or phasic EMG activity above background EMG activity or (ii) In 30 s bins: 27% increase in tonic and/or phasic EMG activity above background.

Data downloaded from www.ppmi-info.org/data on May 1st, 2017 were used for this analysis.

The study was approved by institutional review boards at PPMI sites, and written informed consent was obtained. This trial is registered at ClinicalTrials.gov, Identifier: NCT01141023.

### Assessments

Screening visit assessments included:

- Demographics: age, sex, education, family history of PD in a 1st or 2nd degree relative, handedness.
- Clinical features: RBD symptom duration and RBD disease duration (time from iRBD diagnosis to screening visit).
- MDS-UPDRS I. Items considered in this analysis were those that query non-motor symptoms associated with the prodromal PD state: cognitive impairment (MDS-UPDRS item 1.1), hallucinations (MDS-UPDRS item 1.2), depression (MDS-UPDRS item 1.3), anxiety (MDS-UPDRS item 1.4), and apathy (MDS-UPDRS item 1.5).
- MDS-UPDRS II. It Items considered in this analysis were those that query non-motor symptoms associated with the prodromal PD state:^3,8^ subjective sleep abnormalities (MDS-UPDRS item 1.7), daytime sleepiness (MDS-UPDRS item 1.8), fatigue (MDS-UPDRS item 1.13), constipation (MDS-UPDRS item 1.11), and light-headedness (MDS-UPDRS item 1.12)).
- MDS-UPDRS III: the MDS-UPDRS part III score was the measure of motor function administered. Bradykinesia, rigidity, and tremor subscores were calculated as follows:
  - Total bradykinesia subscore = sum of items 3.4, 3.5, 3.6, 3.7, and 3.8 of MDS-UPDRS part III
  - Total tremor subscore = sum of items 3.15, 3.16, 3.17, and 3.18 of MDS-UPDRS part III
  - Total rigidity subscore = sum of responses on item 3.3 of MDS-UPDRS part III.

- Blood pressure measurement. Orthostatic hypotension = change of 20 mm in systolic and 10 mm in diastolic from supine to standing.
- Montreal Cognitive Assessment (MoCA)^11^: Total score of MOCA and item responses examined as reflection of specific cognitive domains: number of words generated on word list generation (F) test of MOCA; Number of words recalled on MOCA delayed recall; Total score on visuospatial/executive component of MOCA (trails, cube copying, clock drawing).

DATscan SPECT was conducted as previously described.^12–15^ All scans were quantitatively analyzed and visually assessed centrally at the PPMI imaging core lab at the Institute for Neurodegenerative Disorders.

Scans were quantitatively analyzed via extracting count densities for the left caudate, right caudate, left putamen, right putamen, and occipital cortex and calculating specific binding ratios (SBRs) for each of the 4 striatal regions. The minimum putamen (calculated as the minimum SBR value from either the left and right putamen) was determined. A subject was deemed to have a normal scan if lowest putamen binding was >80th %ile expected for age.^12,13^ A subject was deemed to have a DAT binding deficit if the putamen binding was <60% predicted for age. This cutoff has robust predictive value for future risk of PD,^9^ especially among individuals with other prodromal signs/symptoms.^16^ For subjects with binding in the 60th–80th%ile expected for age, visual interpretation (VI) was used to determine study eligibility. Scans were visually assessed by two different expert readers in the field of nuclear medicine for evidence of DAT deficit. Scans were read as either positive or negative. Negative scans are characterized by two relatively symmetric comma or crescent-shaped focal regions of radiotracer uptake mirrored about the midline in transaxial images. Striatal uptake, comprising both the caudate and putamen, is distinctly evident compared to surrounding brain tissue. Positive scans typically fall into at least one of the following three general categories: (i) Activity is asymmetric, e.g., uptake in the region of the putamen of one hemisphere is absent or greatly reduced with respect to the other. Uptake is still visible in the caudate nuclei of both hemispheres resulting in a comma or crescent shape in one and a circular or oval focus in the other. There may be reduced uptake between at least one striatum and surrounding tissues. (ii) DAT uptake is absent in the putamen of both hemispheres and confined to the caudate nuclei. The signal is relatively symmetric and forms two roughly circular or oval foci. Uptake of one or both is generally reduced. (iii) Uptake is absent in the putamen of both hemispheres and greatly reduced in one or both caudate nuclei. Uptake of the striata with respect to the background is reduced (see also DaTSCAN™ [package insert]. Arlington Heights, IL: GE Healthcare; 2011).

The following continuous measures of DAT binding were also examined in this analysis: (i) left and right putamen SBR (ii) left and right caudate SBR, (iii) mean striatal SBR (average of putamen and caudate SBR on right and left) (iv) mean putamen binding (average of right and left putamen binding) (v) mean caudate binding (average of right and left caudate binding) (vi) Putamen Asymmetry Index, defined as = (left putamen binding-right putamen binding)/((left putamen + right putamen binding)/2) * 100 (vii) Caudate Asymmetry Index, defined as = (left caudate binding-right caudate binding)/((left caudate + right caudate binding)/2) * 100.

### Statistical analysis

Analyses were performed using SAS 9.4 (SAS Institute Inc., Cary, NC). *T*-tests, Chi-square, and Fisher’s exact test as appropriate were used to compare baseline demographics, clinical features, and DAT binding variables between groups. For non-motor symptoms measured on MDS-UPDRS items, a response of >0 was given if the symptom was considered present. Additionally, sensitivity analyses were conducted where a score of >1 was required.

Logistic regression models were used to examine demographic and clinical predictors of DAT binding deficit (as a binary outcome) in iRBD subjects. Linear models were used to examine relationships between demographic and clinical predictors and DAT binding variables as continuous outcomes. The univariate relationship of each predictor with the outcome was examined. Any variables with univariate associations with *p*-values < 0.20 were included in a multivariable model. All models were adjusted for age and disease duration.

### Code availability

The code used to generate and analyze the dataset utilized in this analysis are available, in SAS 9.4 format, upon request.

## Supplementary information

Supplementary Information

## Data Availability

DATscan SPECT results for the prodromal cohort of PPMI are not publicly available. They are sequestered and only available to the statistics core who conducted these analyses. All other data for the discovery cohort (PPMI) used in these analyses are available for download at ppmi-info.org.

## Supplementary information

**Supplementary information** accompanies the paper on the *npj Parkinson’s Disease* website (10.1038/s41531-018-0073-1).
