# Supplementary material for: Basic clinical features do not predict dopamine transporter binding in idiopathic REM behavior disorder
Source: NPJ Parkinsons Dis. 2019 Jan 29;5:2. doi: 10.1038/s41531-018-0073-1 (PMC6351563; doi:10.1038/s41531-018-0073-1)
Supplement: Supplementary file 1 — Supplementary Information [file 41531_2018_73_MOESM1_ESM.docx]

**Supplementary table 1.** Differences in MDS-UPDRS responses using cutoff value of 0-1 vs. >1 on individual item responses, in the iRBD group with DAT binding deficit compared to the iRBD without DAT binding deficit

|  | **Screened RBD Subjects** | | |
| --- | --- | --- | --- |
|  | **DAT Deficit+** | **Dat Deficit-** | **p-value** |
| **Variable** | **(N=49)** | **(N=26)** | **(DAT+ vs DAT-)** |
| **MDS-UPDRS I Cognitive Impairment** |  |  | 1.0000 |
| 0-1 | 46 (93.88%) | 25 (96.15%) |  |
| >1 | 3 (6.12%) | 1 (3.85%) |  |
| Missing | 0 (0%) | 0 (0%) |  |
| **MDS-UPDRS I Hallucinations** |  |  | N/A |
| 0-1 | 49 (100.00%) | 26 (100.00%) |  |
| >1 | 0 (0%) | 0 (0%) |  |
| Missing | 0 (0%) | 0 (0%) |  |
| **MDS-UPDRS I Depression** |  |  | 1.0000 |
| 0-1 | 42 (85.71%) | 22 (84.62%) |  |
| >1 | 7 (14.29%) | 4 (15.38%) |  |
| Missing | 0 (0%) | 0 (0%) |  |
| **MDS-UPDRS I Anxiety** |  |  | 0.6878 |
| 0-1 | 45 (91.84%) | 23 (88.46%) |  |
| >1 | 4 (8.16%) | 3 (11.54%) |  |
| Missing | 0 (0%) | 0 (0%) |  |
| **MDS-UPDRS I Apathy** |  |  | N/A |
| 0-1 | 49 (100.00%) | 26 (100.00%) |  |
| >1 | 0 (0%) | 0 (0%) |  |
| Missing | 0 (0%) | 0 (0%) |  |
| **MDS-UPDRS I Sleep Abnormalities** |  |  | 1.0000 |
| 0-1 | 15 (30.61%) | 8 (30.77%) |  |
| >1 | 34 (69.39%) | 18 (69.23%) |  |
| Missing | 0 (0%) | 0 (0%) |  |
| **MDS-UPDRS I Daytime Sleepiness** |  |  | 0.8112 |
| 0-1 | 28 (57.14%) | 14 (53.85%) |  |
| >1 | 21 (42.86%) | 12 (46.15%) |  |
| Missing | 0 (0%) | 0 (0%) |  |
| **MDS-UPDRS I Urinary Problems** |  |  | 1.0000 |
| 0-1 | 42 (85.71%) | 22 (84.62%) |  |
| >1 | 7 (14.29%) | 4 (15.38%) |  |
| Missing | 0 (0%) | 0 (0%) |  |
| **MDS-UPDRS I Light Headedness** |  |  | 1.0000 |
| 0-1 | 43 (87.76%) | 23 (88.46%) |  |
| >1 | 6 (12.24%) | 3 (11.54%) |  |
| Missing | 0 (0%) | 0 (0%) |  |
| **MDS-UPDRS I Fatigue** |  |  | 0.3543 |
| 0-1 | 42 (85.71%) | 20 (76.92%) |  |
| >1 | 7 (14.29%) | 6 (23.08%) |  |
| Missing | 0 (0%) | 0 (0%) |  |
| **MDS-UPDRS I Constipation** |  |  | 0.3872 |
| 0-1 | 36 (73.47%) | 22 (84.62%) |  |
| >1 | 13 (26.53%) | 4 (15.38%) |  |
| Missing | 0 (0%) | 0 (0%) |  |
| **MDS-UPDRS II Tremor** |  |  | 0.5472 |
| 0-1 | 46 (93.88%) | 26 (100.00%) |  |
| >1 | 3 (6.12%) | 0 (0.00%) |  |
| Missing | 0 (0%) | 0 (0%) |  |
| **MDS-UPDRS II Walking and Balance** |  |  | 1.0000 |
| 0-1 | 47 (95.92%) | 25 (96.15%) |  |
| >1 | 2 (4.08%) | 1 (3.85%) |  |
| Missing | 0 (0%) | 0 (0%) |  |
| **MDS-UPDRS III Total Score** |  |  | 0.7023 |
| Mean (SD) | 4.51 (3.8) | 4.15 (3.9) |  |
| (Min, Max) | (0.0, 15.0) | (0.0, 13.0) |  |
| Missing | 0 | 0 |  |
| **Right MDS-UPDRS III Total Score** |  |  | 0.8865 |
| Mean (SD) | 1.24 (1.5) | 1.19 (1.5) |  |
| (Min, Max) | (0.0, 5.0) | (0.0, 5.0) |  |
| Missing | 0 | 0 |  |
| **Left MDS-UPDRS III Total Score** |  |  | 0.3358 |
| Mean (SD) | 1.39 (1.4) | 1.77 (1.9) |  |
| (Min, Max) | (0.0, 5.0) | (0.0, 8.0) |  |
| Missing | 0 | 0 |  |
| **Right MDS-UPDRS III Rigidity** |  |  | N/A |
| 0-1 | 49 (100.00%) | 26 (100.00%) |  |
| >1 | 0 (0%) | 0 (0%) |  |
| Missing | 0 (0%) | 0 (0%) |  |
| **Right MDS-UPDRS III Bradykinesia** |  |  | 0.3543 |
| 0-1 | 42 (85.71%) | 20 (76.92%) |  |
| >1 | 7 (14.29%) | 6 (23.08%) |  |
| Missing | 0 (0%) | 0 (0%) |  |
| **Right MDS-UPDRS III Tremor** |  |  | 1.0000 |
| 0-1 | 42 (85.71%) | 23 (88.46%) |  |
| >1 | 7 (14.29%) | 3 (11.54%) |  |
| Missing | 0 (0%) | 0 (0%) |  |
| **Left MDS-UPDRS III Rigidity** |  |  | N/A |
| 0-1 | 49 (100.00%) | 26 (100.00%) |  |
| >1 | 0 (0%) | 0 (0%) |  |
| Missing | 0 (0%) | 0 (0%) |  |
| **Left MDS-UPDRS III Bradykinesia** |  |  | 0.4403 |
| 0-1 | 35 (71.43%) | 16 (61.54%) |  |
| >1 | 14 (28.57%) | 10 (38.46%) |  |
| Missing | 0 (0%) | 0 (0%) |  |
| **Left MDS-UPDRS III Tremor** |  |  | 0.7105 |
| 0-1 | 44 (89.80%) | 22 (84.62%) |  |
| >1 | 5 (10.20%) | 4 (15.38%) |  |
| Missing | 0 (0%) | 0 (0%) |  |

**Supplementary Table 2**. Results of logistic regression model examining predictors of DAT reduction. Analyses adjust for age and disease duration

|  |  | **# Observations** | **OR Estimate** | **Multivariate** |
| --- | --- | --- | --- | --- |
| **Variable** | **p-value** | **Missing** | **(95% CI)** | **p-value** |
| Gender | 0.2349 | 4 | - | - |
| Education | 0.2391 | 4 | - | - |
| Family History of PD | 0.3956 | 4 | - | - |
| Handedness | 0.9054 | 4 | - | - |
| Orthostatic hypotension | 0.0823 | 4 | 0.367 (0.120, 1.125) | 0.0796 |
| MOCA | 0.5425 | 4 | - | - |
| MOCA Word Generation | 0.8481 | 4 | - | - |
| MOCA Delayed Recall | 0.4370 | 4 | - | - |
| MOCA Visuospatial Total Score | 0.7961 | 4 | - | - |
| MDS-UPDRS I Cognitive Impairment | 0.3812 | 4 | - | - |
| MDS-UPDRS I Hallucinations | 0.2002 | 4 | - | - |
| MDS-UPDRS I Depression | 0.1025 | 4 | 3.299 (0.950, 11.45) | 0.0602 |
| MDS-UPDRS I Anxiety | 0.9978 | 4 | - | - |
| MDS-UPDRS I Apathy | 0.1779 | 4 | 0.121 (0.012, 1.250) | 0.0763 |
| MDS-UPDRS II Sleep Abnormalities | 0.2420 | 4 | - | - |
| MDS-UPDRS II Fatigue | 0.3863 | 4 | - | - |
| MDS-UPDRS II Constipation | 0.3561 | 4 | - | - |
| MDS-UPDRS III Total Score | 0.6949 | 4 | - | - |
| Right MDS-UPDRS III Total Score | 0.9646 | 4 | - | - |
| Left MDS-UPDRS III Total Score | 0.4720 | 4 | - | - |
| Right MDS-UPDRS III Rigidity | 0.5851 | 4 | - | - |
| Right MDS-UPDRS III Bradykinesia | 0.4916 | 4 | - | - |
| Right MDS-UPDRS III Tremor | 0.4021 | 4 | - | - |
| Left MDS-UPDRS III Rigidity | 0.4366 | 4 | - | - |
| Left MDS-UPDRS III Bradykinesia | 0.5636 | 4 | - | - |
| Left MDS-UPDRS III Tremor | 0.6937 | 4 | - | - |

**Supplementary table 3**. Results of linear regression models examining the predictors of mean striatum specific binding ratio. Analyses adjust for age and disease duration.

|  |  | **# Observations** |  |
| --- | --- | --- | --- |
| **Variable** | **p-value** | **Missing** | **Multivariable p-value** |
| Handedness | 0.3328 | 0 | - |
| Right MDS-UPDRS III Bradykinesia | 0.4711 | 0 | - |
| Right MDS-UPDRS III Rigidity | 0.2963 | 0 | - |
| Right MDS-UPDRS III Tremor | 0.5453 | 0 | - |
| Left MDS-UPDRS III Bradykinesia | 0.9583 | 0 | - |
| Left MDS-UPDRS III Rigidity | 0.2728 | 0 | - |
| Left MDS-UPDRS III Tremor | 0.3398 | 0 | - |
| MDS-UPDRS III Total Score | 0.1421 | 0 | NS |
| MOCA | 0.7524 | 0 | - |
| MDS-UPDRS I Total Score | 0.9232 | 0 | - |
| MDS-UPDRS II Total Score | 0.6614 | 0 | - |
| MDS-UPDRS I+II Total Score | 0.8733 | 0 | - |
